# Supplementary material for: Structural conservation of antibiotic interaction with ribosomes
Source: Nat Struct Mol Biol. 2023 Aug 7;30(9):1380–92. doi: 10.1038/s41594-023-01047-y (PMC10497419; doi:10.1038/s41594-023-01047-y)
Supplement: Supplementary file 1 — Supplementary Figs. 1–18, Table 1 and References. [file 41594_2023_1047_MOESM1_ESM.pdf]

nature structural & molecular biology

Article https://doi.org/10.1038/s41594-023-01047-y

Structural conservation of antibiotic interaction with ribosomes

In the format provided by the authors and unedited

Table of Contents

Content	Page
Supplementary Figure 1: Tetracycline	2
Supplementary Figure 2: Omadacycline	3
Supplementary Figure 3: Eravacycline	4
Supplementary Figure 4: Pentacycline	5
Supplementary Figure 5: Hygromycin B	6
Supplementary Figure 6: Gentamicin	7
Supplementary Figure 7: Spectinomycin	8
Supplementary Figure 8: Streptomycin	9
Supplementary Figure 9: Apramycin	10
Supplementary Figure 10: Kasugamycin	11
Supplementary Figure 11: Capreomycin	12
Supplementary Figure 12: Avilamycin	13
Supplementary Figure 13: Evernimicin	14
Supplementary Figure 14: Tiamulin	15
Supplementary Figure 15: Retapamulin	16
Supplementary Figure 16: Lincomycin	17
Supplementary Figure 17: Clindamycin	18
Supplementary Figure 18: Representative micrographs	19
Supplementary Table 1: Summary of antibiotic-ribosome structures	20
Supplementary References	25

Supplementary Figures

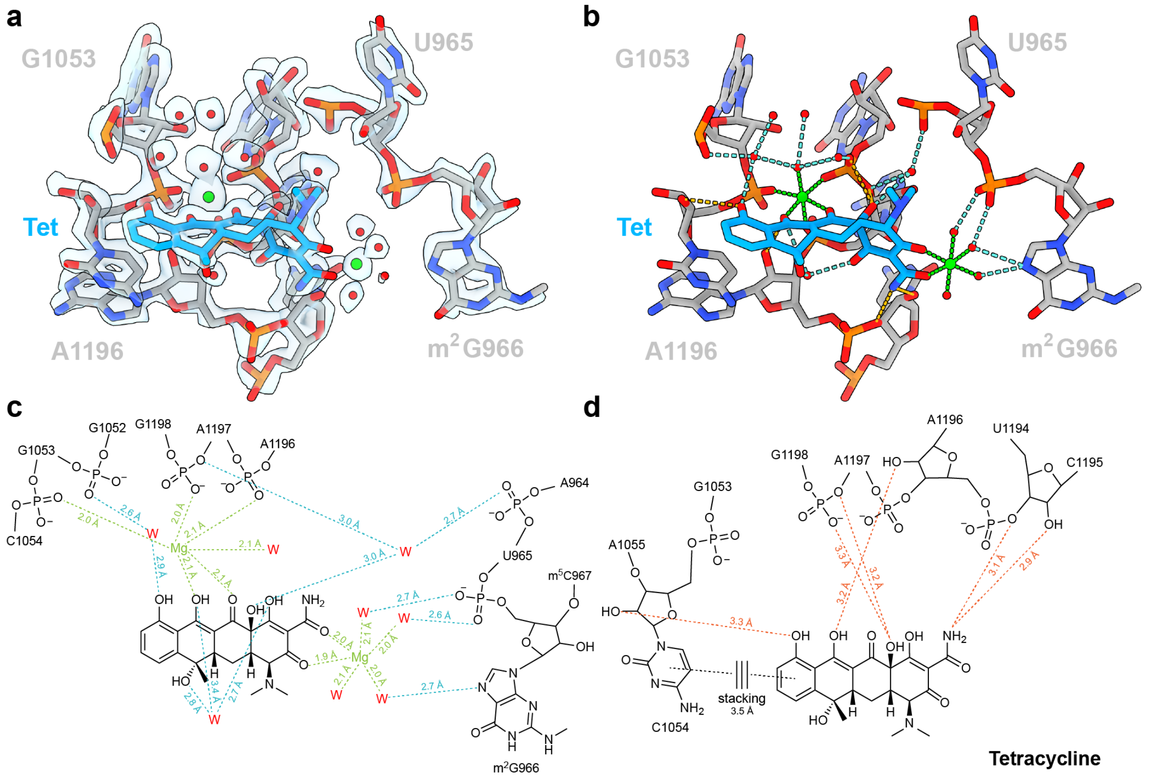

Supplementary Fig. 1 | Interaction of tetracycline on the SSU-head. a, cryo-EM density (transparent surface) for drug and nucleotides (not for solvent) with molecule model for tetracycline (blue), 16S rRNA nucleotides (grey), water molecules (red spheres) and Mg ions (green spheres). **b,** same view as (**a**) without density but with dashed lines indicating direct (orange) or indirect water (blue) or Mg ion (green) mediated interactions. **c-d,** schematic representation of the (**c**) indirect water (blue) or Mg ion (green) and (**d**) direct (orange) interactions between tetracycline and the SSU-head.

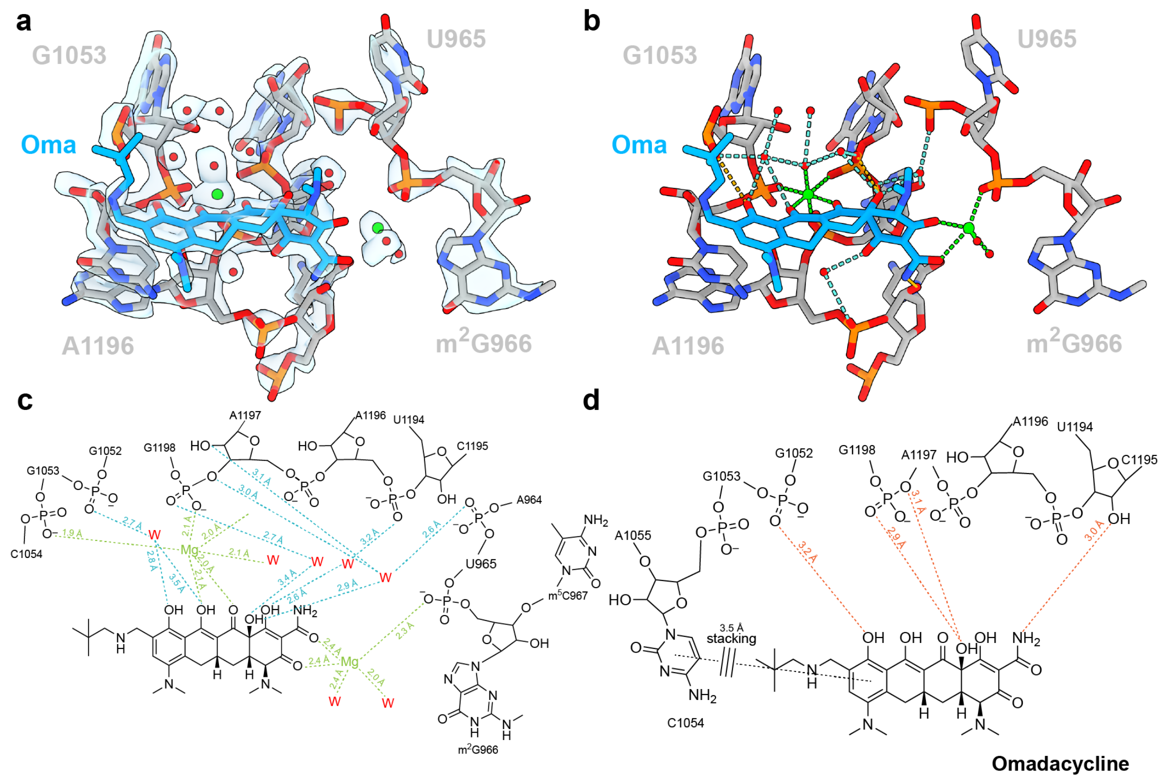

Supplementary Fig. 2 | Interaction of omadacycline on the SSU-head. a, cryo-EM density (transparent surface) for drug and nucleotides (not for solvent) with molecule model for omadacycline (blue), 16S rRNA nucleotides (grey), water molecules (red spheres) and Mg ions (green spheres). **b,** same view as (**a**) without density but with dashed lines indicating direct (orange) or indirect water (blue) or Mg ion (green) mediated interactions. **c-d,** schematic representation of the (**c**) indirect water (blue) or Mg ion (green) and (**d**) direct (orange) interactions between omadacycline and the SSU head.

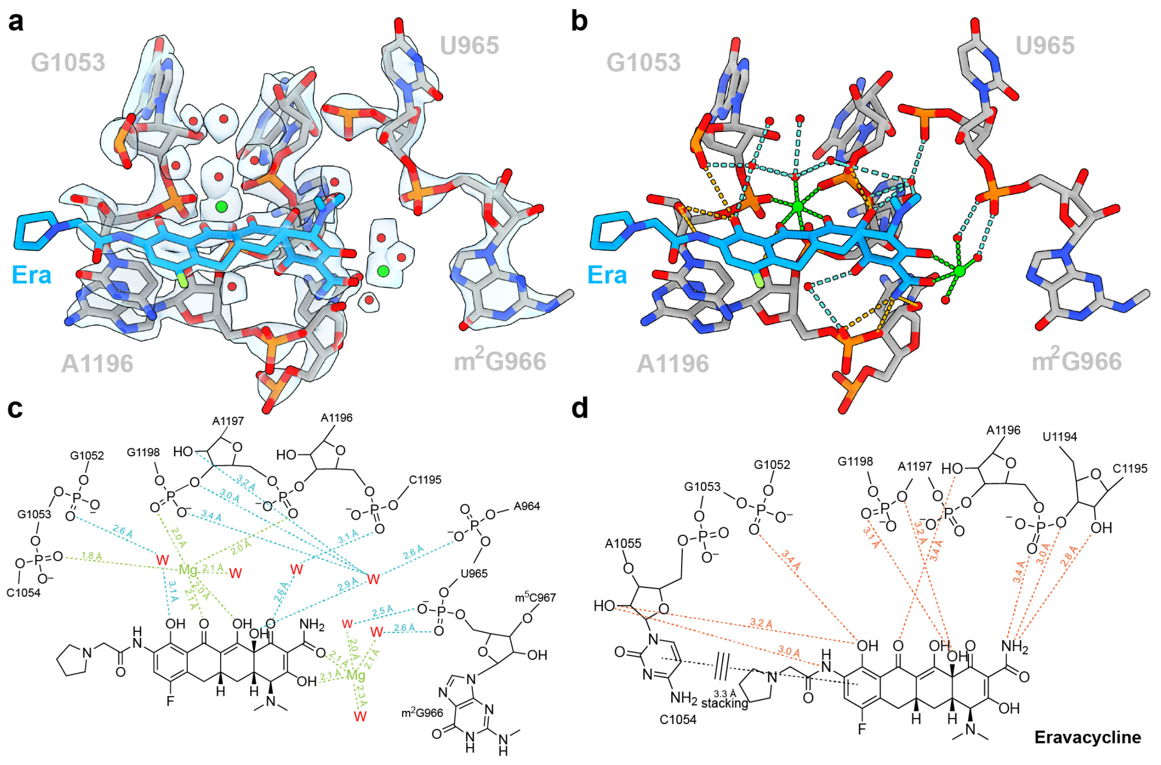

Supplementary Fig. 3 | Interaction of eravacycline on the SSU-head. a, cryo-EM density (transparent surface) for drug and nucleotides (not for solvent) with molecule model for eravacycline (blue), 16S rRNA nucleotides (grey), water molecules (red spheres) and Mg ions (green spheres). **b,** same view as (**a**) without density but with dashed lines indicating direct (orange) or indirect water (blue) or Mg ion (green) mediated interactions. **c-d,** schematic representation of the (**c**) indirect water (blue) or Mg ion (green) and (**d**) direct (orange) interactions between eravacycline and the SSU head.

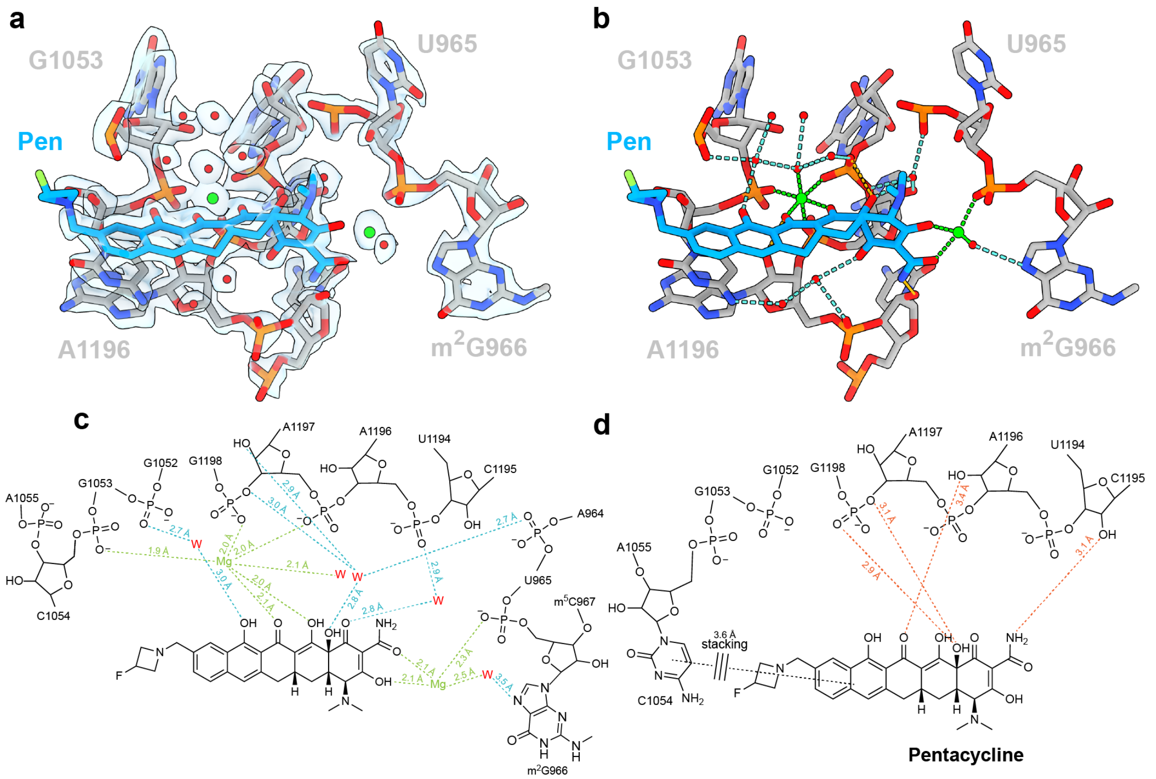

Supplementary Fig. 4 | Interaction of pentacycline on the SSU-head. a, cryo-EM density (transparent surface) for drug and nucleotides (not for solvent) with molecule model for pentacycline (blue), 16S rRNA nucleotides (grey), water molecules (red spheres) and Mg ions (green spheres). **b,** same view as (**a**) without density but with dashed lines indicating direct (orange) or indirect water (blue) or Mg ion (green) mediated interactions. **c-d,** schematic representation of the (**c**) indirect water (blue) or Mg ion (green) and (**d**) direct (orange) interactions between pentacycline and the SSU head.

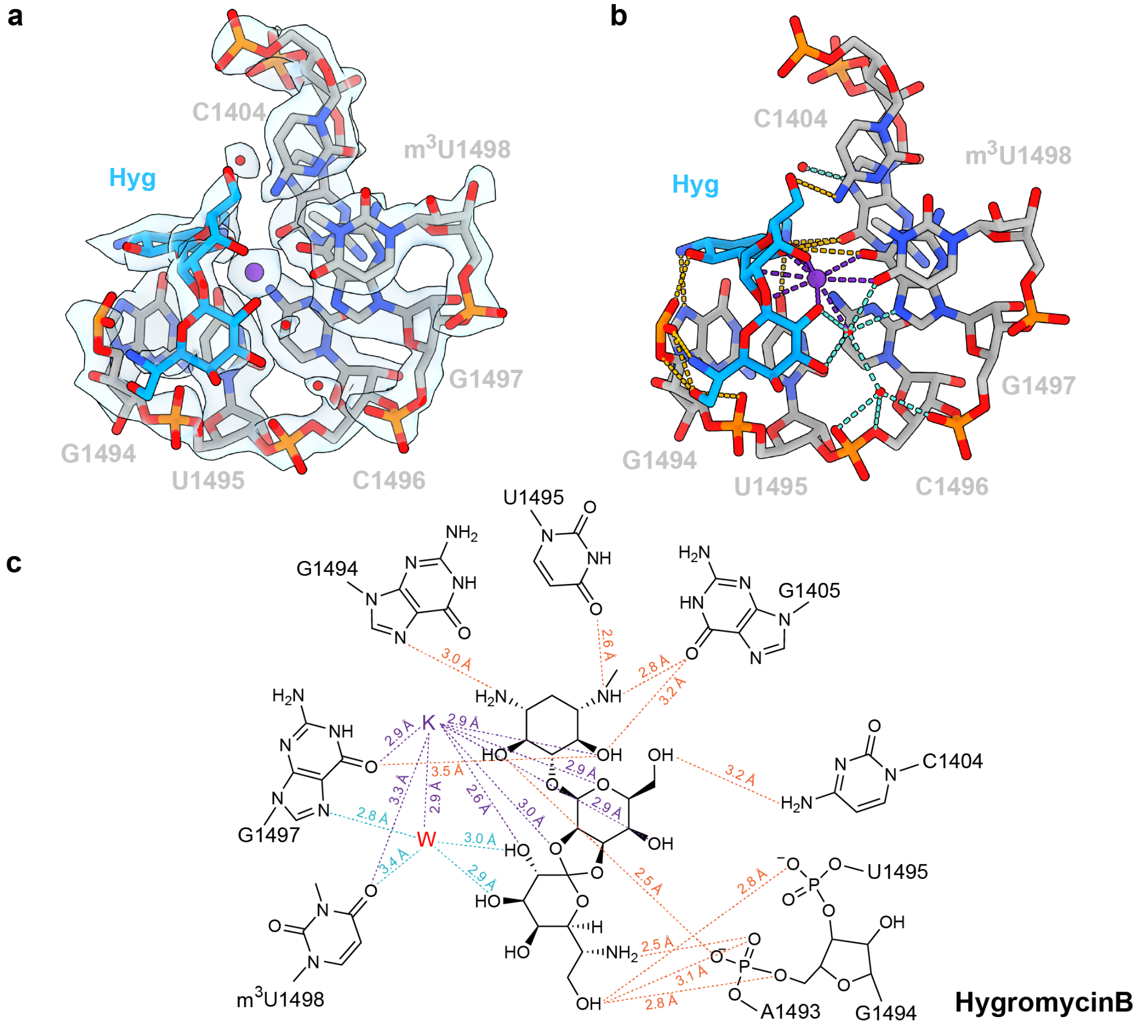

Supplementary Fig. 5 | Interaction of hygromycin B on the SSU-body. a, cryo-EM density (transparent surface) for drug and nucleotides (not for solvent) with molecule model for hygromycin B (blue), 16S rRNA nucleotides (grey), water molecules (red spheres) and a putative K^+ ion (purple sphere). **b,** same view as (**a**) without density but with dashed lines indicating direct (orange) or indirect water (blue) or a putative K^+ ion (purple) mediated interactions. **c,** schematic representation of the indirect water (blue) or a putative K^+ ion (purple) as well as direct (orange) interactions between hygromycin B and the SSU body.

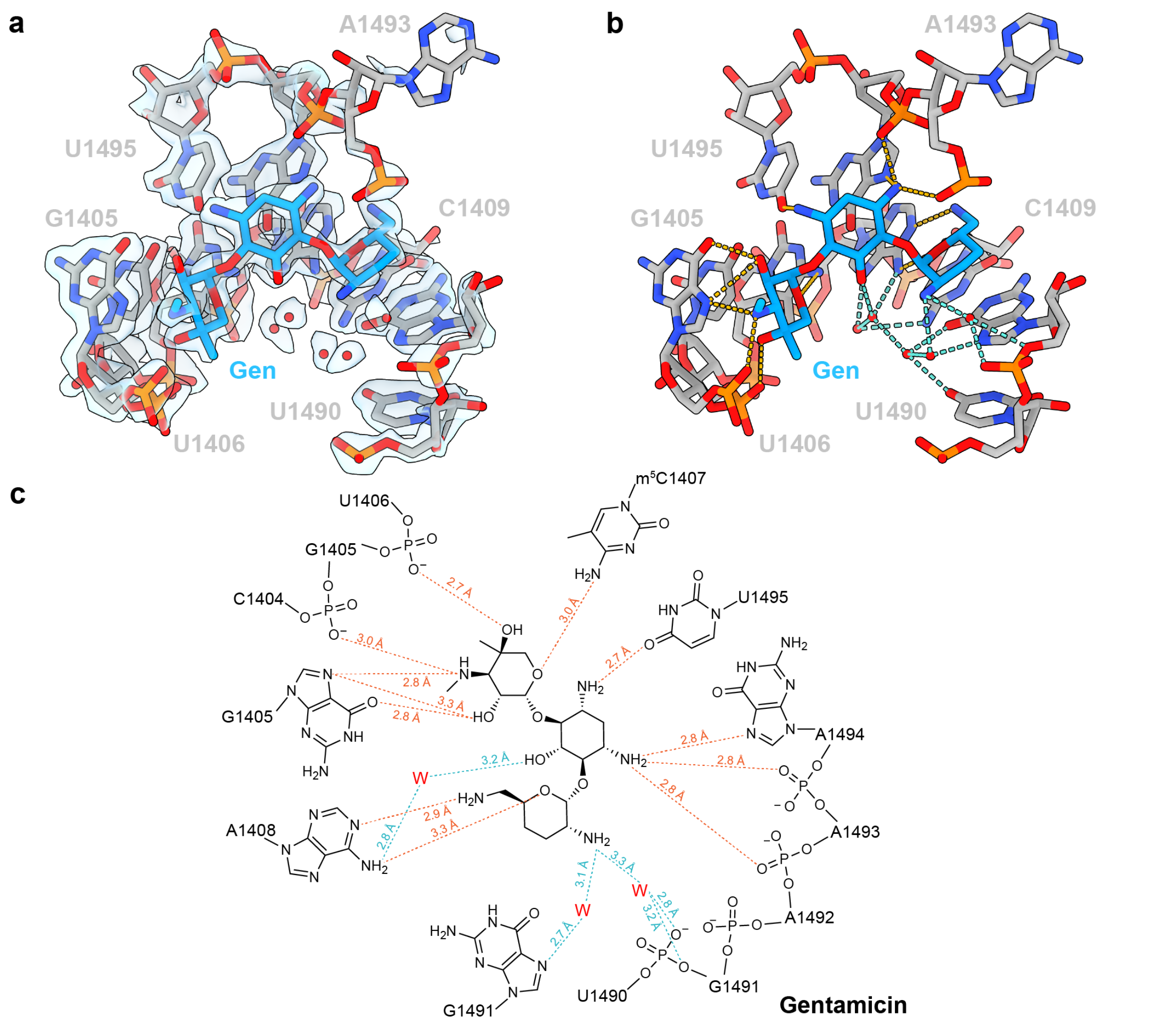

Supplementary Fig. 6 | Interaction of gentamicin on the SSU-body. a, cryo-EM density (transparent surface) for drug and nucleotides (not for solvent) with molecule model for gentamicin (blue), 16S rRNA nucleotides (grey) and water molecules (red spheres). **b,** same view as (**a**) without density but with dashed lines indicating direct (orange) or indirect (blue) water-mediated interactions. **c,** schematic representation of the indirect water (blue) and direct (orange) interactions between gentamicin and the SSU body.

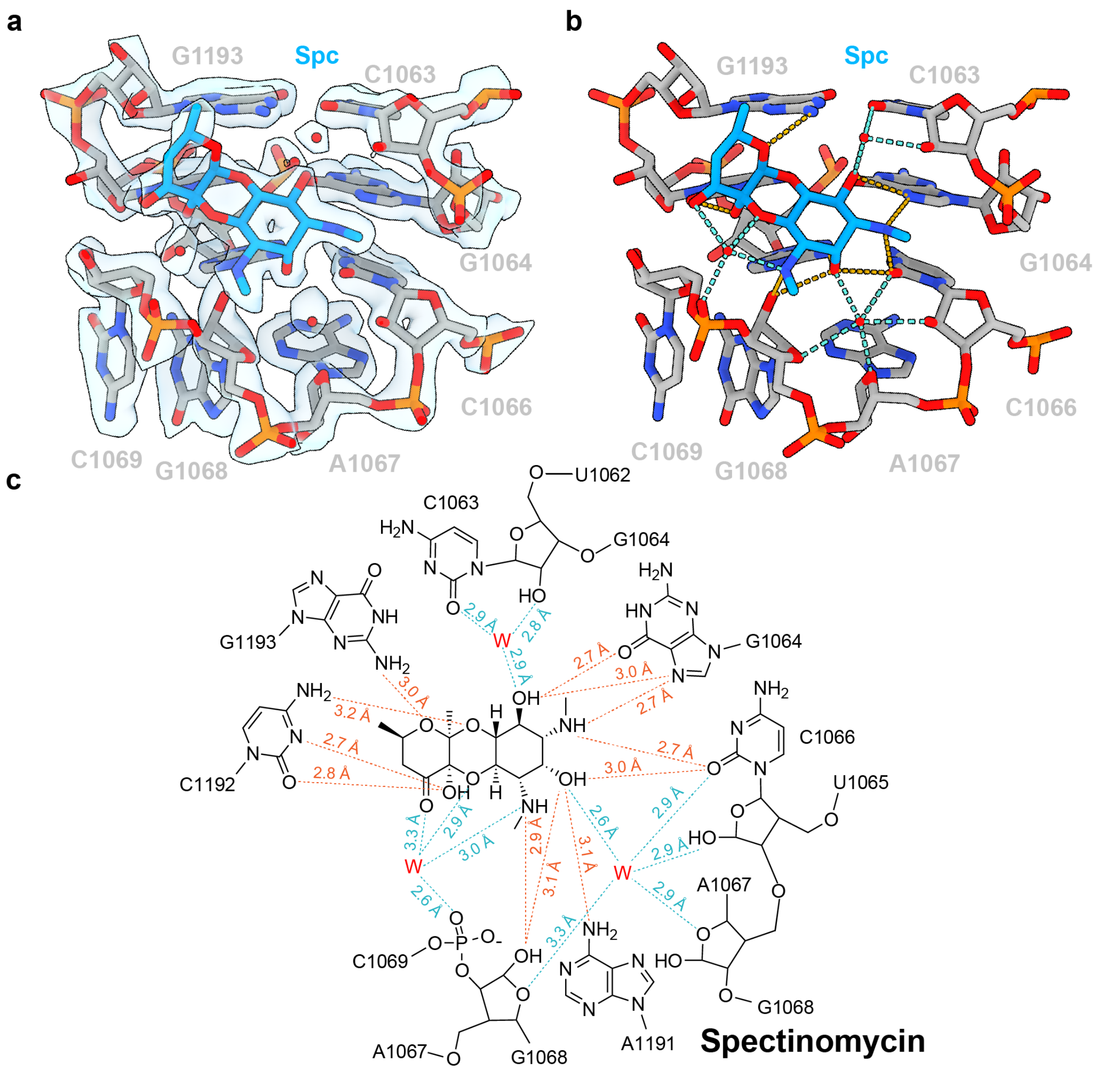

Supplementary Fig. 7 | Interaction of spectinomycin on the SSU. a, cryo-EM density (transparent surface) for drug and nucleotides (not for solvent) with molecule model for spectinomycin (blue), 16S rRNA nucleotides (grey) and water molecules (red spheres). **b,** same view as (**a**) without density but with dashed lines indicating direct (orange) or indirect (blue) water-mediated interactions. **c,** schematic representation of the indirect water (blue) and direct (orange) interactions between spectinomycin and the SSU.

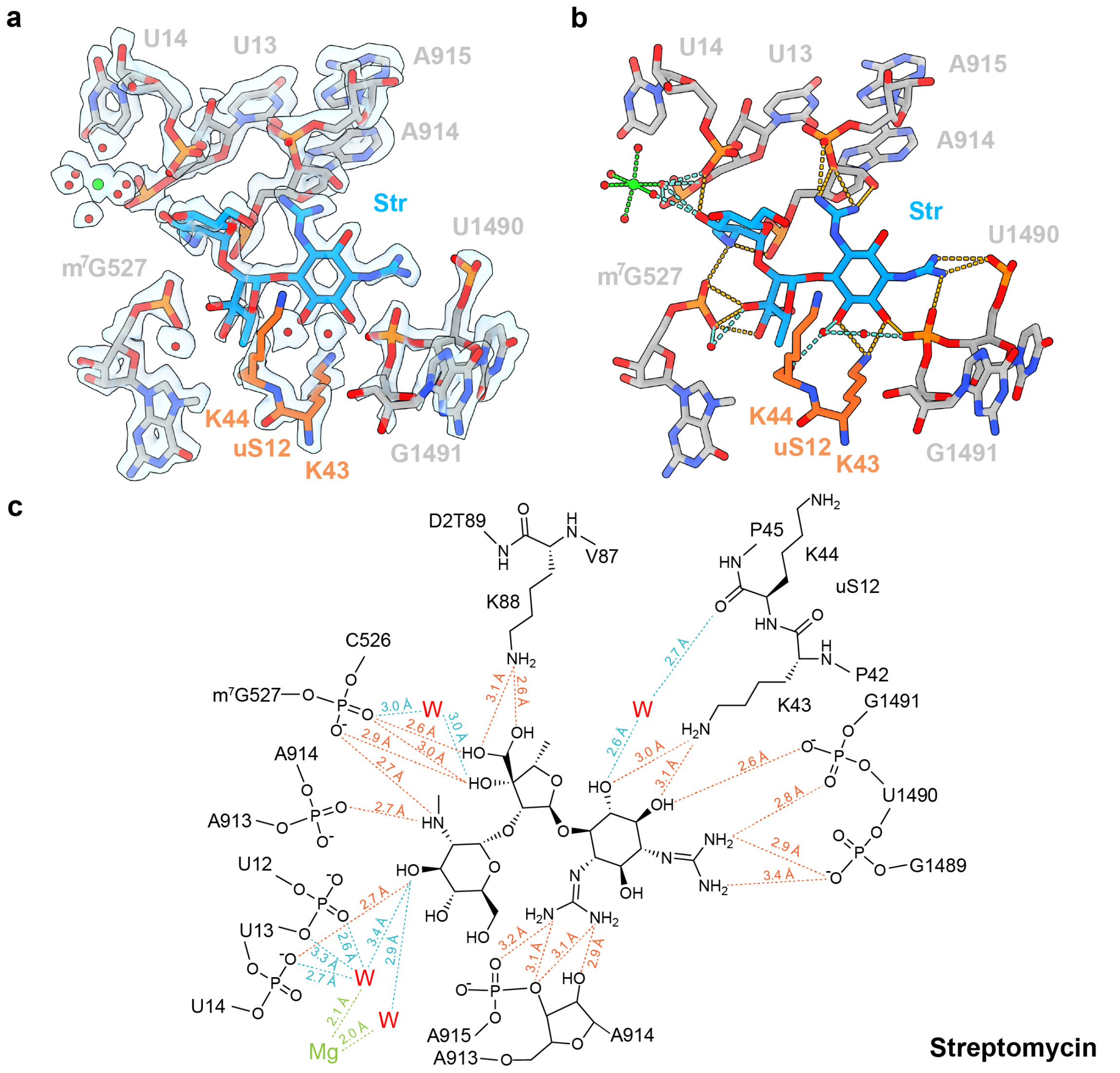

Supplementary Fig. 8 | Interaction of streptomycin on the SSU-body. a, cryo-EM density (transparent surface) for drug and nucleotides (not for solvent) with molecule model for streptomycin (blue), 16S rRNA nucleotides (grey) and water molecules (red spheres). **b,** same view as (**a**) without density but with dashed lines indicating direct (orange) or indirect (blue) water-mediated interactions. **c,** schematic representation of the indirect water (blue) and direct (orange) interactions between streptomycin and the SSU body. Note that in panel a-b, the interaction with K88 of uS12 is not included.

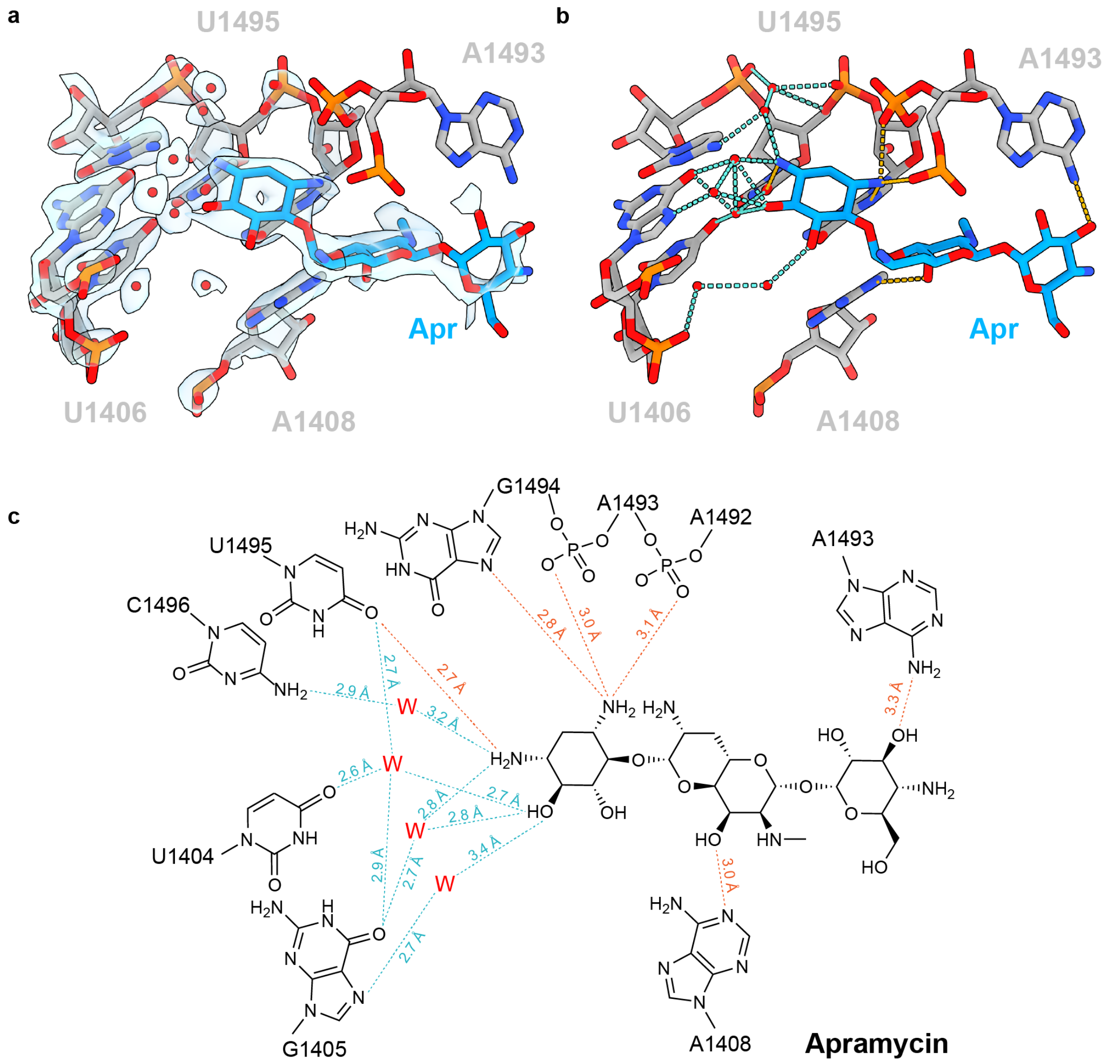

Supplementary Fig. 9 | Interaction of apramycin on the SSU-body. a, cryo-EM density (transparent surface) for drug and nucleotides (not for solvent) with molecule model for apramycin (blue), 16S rRNA nucleotides (grey) and water molecules (red spheres). **b,** same view as (**a**) without density but with dashed lines indicating direct (orange) or indirect (blue) water-mediated interactions. **c,** schematic representation of the indirect water (blue) and direct (orange) interactions between apramycin and the SSU body.

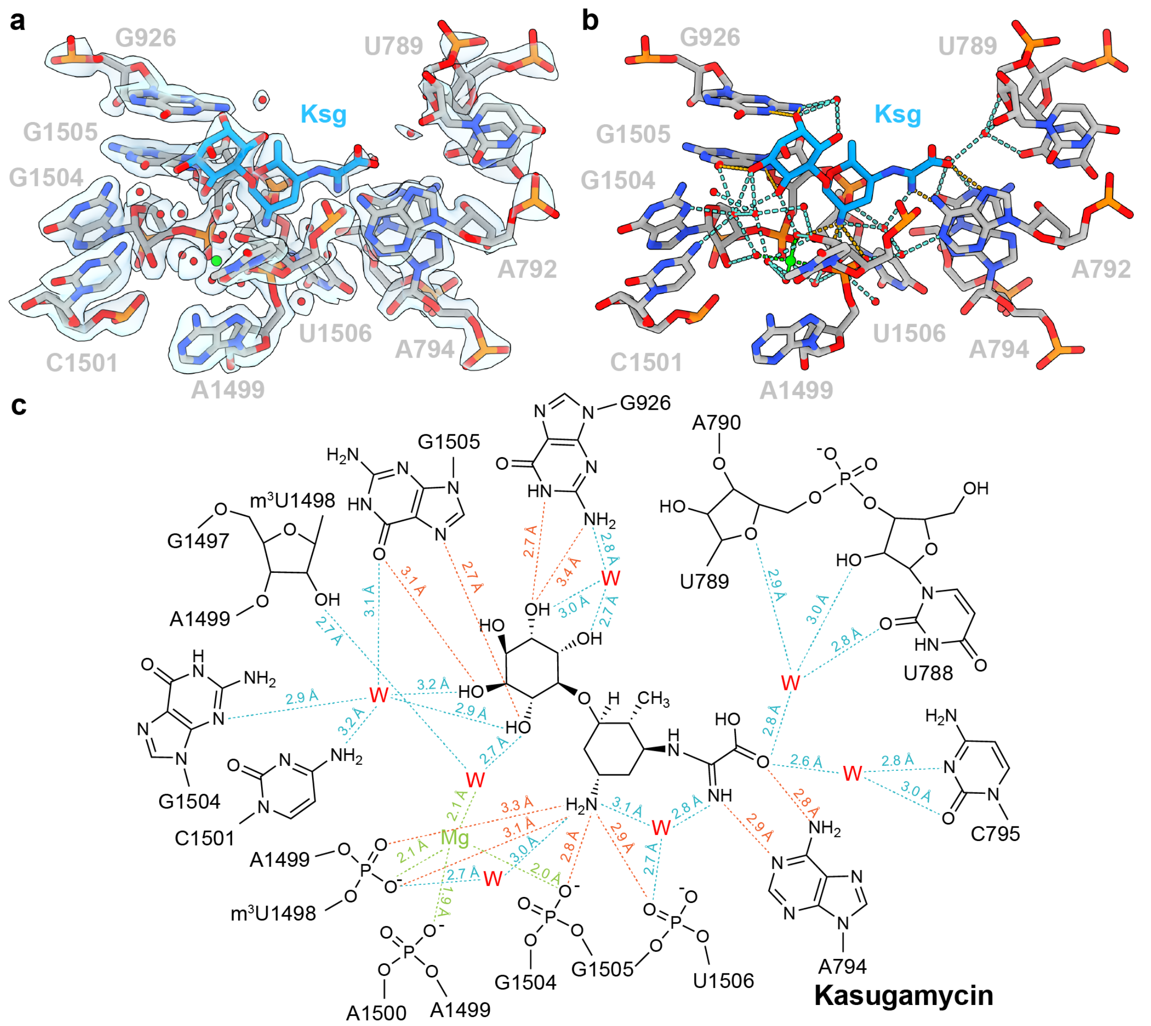

Supplementary Fig. 10 | Interaction of kasugamycin on the SSU-body. a, cryo-EM density (transparent surface) for drug and nucleotides (not for solvent) with molecule model for kasugamycin (blue), 16S rRNA nucleotides (grey) and water molecules (red spheres). **b,** same view as (**a**) without density but with dashed lines indicating direct (orange) or indirect (blue) water-mediated interactions. **c,** schematic representation of the indirect water (blue) and direct (orange) interactions between kasugamycin and the SSU body.

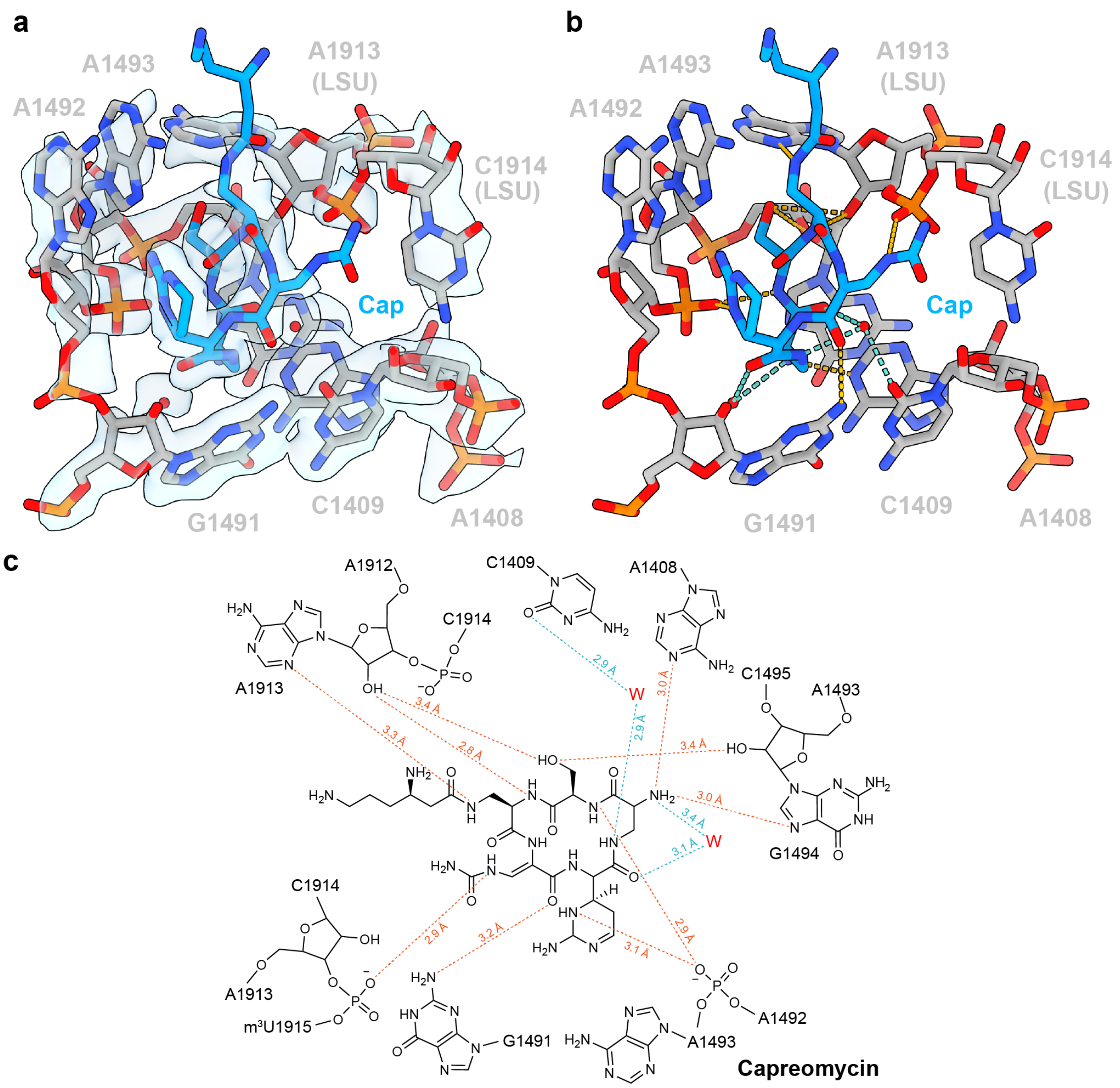

Supplementary Fig. 11 | Interaction of capreomycin at the interface between the SSU and LSU. a, cryo-EM density (transparent surface) for drug and nucleotides (not for solvent) with molecule model for capreomycin (blue), 16S/23S rRNA nucleotides (grey) and water molecules (red spheres). **b,** same view as (**a**) without density but with dashed lines indicating direct (orange) or indirect (blue) water-mediated interactions. **c,** schematic representation of the indirect water (blue) and direct (orange) interactions between capreomycin and the SSU/LSU.

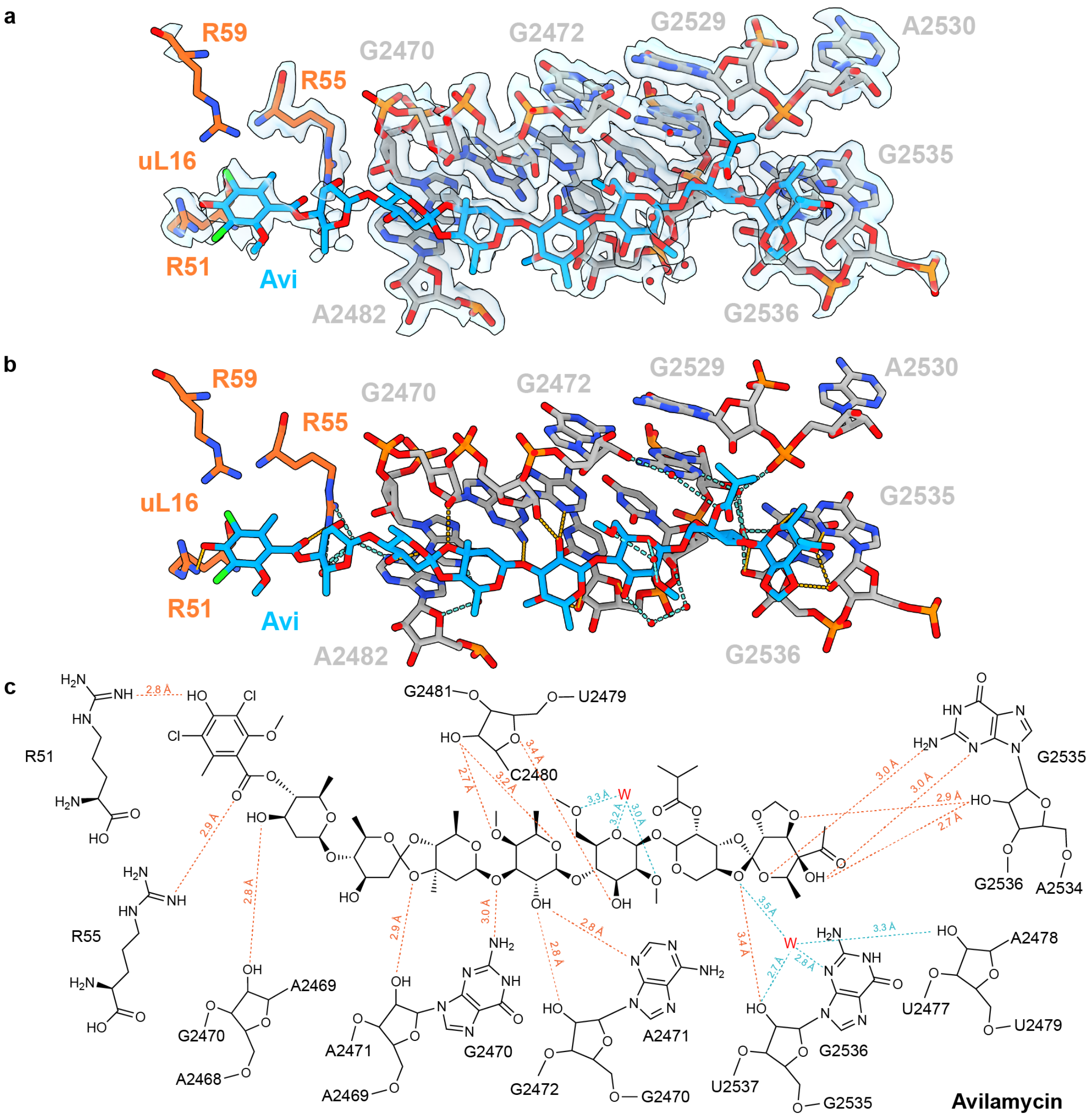

Supplementary Fig. 12 | Interaction of avilamycin on the LSU. a, cryo-EM density (transparent surface) for drug and nucleotides (not for solvent) with molecule model for avilamycin (blue), 23S rRNA nucleotides (grey) and water molecules (red spheres). **b,** same view as (**a**) without density but with dashed lines indicating direct (orange) or indirect (blue) water-mediated interactions. **c,** schematic representation of the indirect water (blue) and direct (orange) interactions between avilamycin and the LSU.

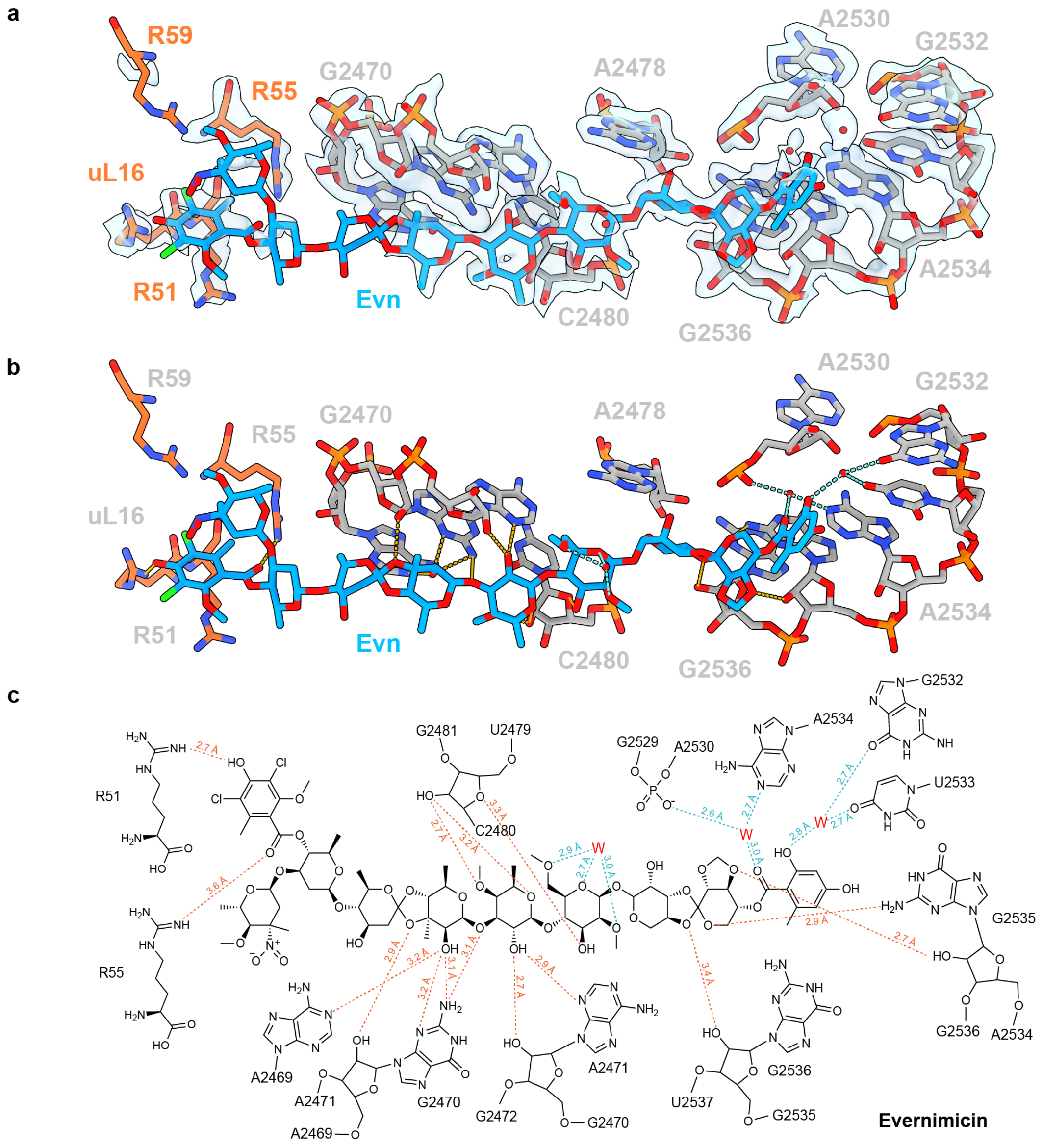

Supplementary Fig. 13 | Interaction of evernimicin on the LSU. a, cryo-EM density (transparent surface) for drug and nucleotides (not for solvent) with molecule model for evernimicin (blue), 23S rRNA nucleotides (grey) and water molecules (red spheres). **b,** same view as (**a**) without density but with dashed lines indicating direct (orange) or indirect (blue) water-mediated interactions. **c,** schematic representation of the indirect water (blue) and direct (orange) interactions between evernimicin and the LSU.

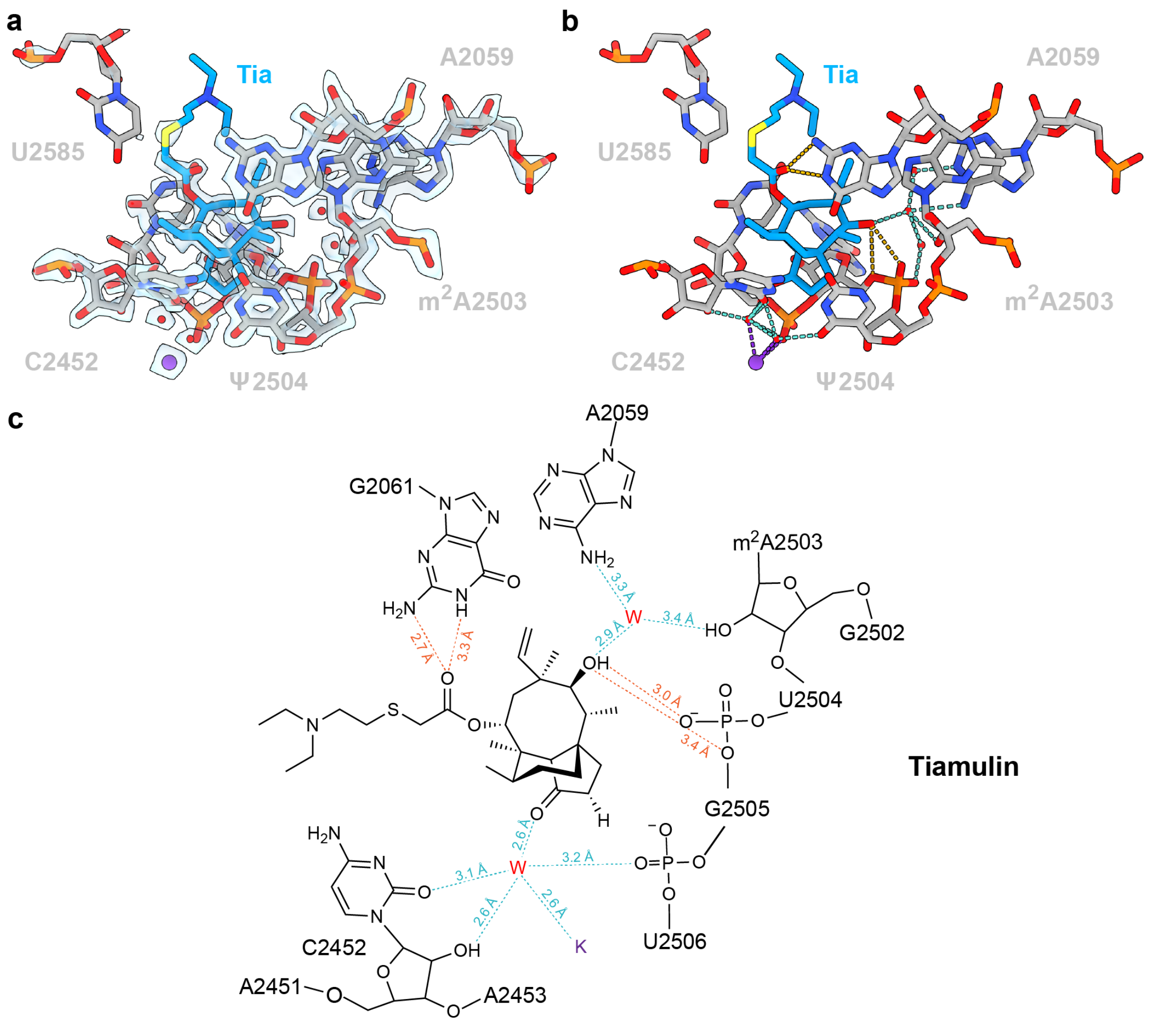

Supplementary Fig. 14 | Interaction of tiamulin on the LSU. a, cryo-EM density (transparent surface) for drug and nucleotides (not for solvent) with molecule model for tiamulin (blue), 23S rRNA nucleotides (grey) and water molecules (red spheres). **b,** same view as (**a**) without density but with dashed lines indicating direct (orange) or indirect (blue) water-mediated and interactions. **c,** schematic representation of the indirect water (blue) and direct (orange) interactions between tiamulin and the LSU, with water (red W) and putative K+ ion (purple K) indicated. The putative K+ ion was assigned based on [1].

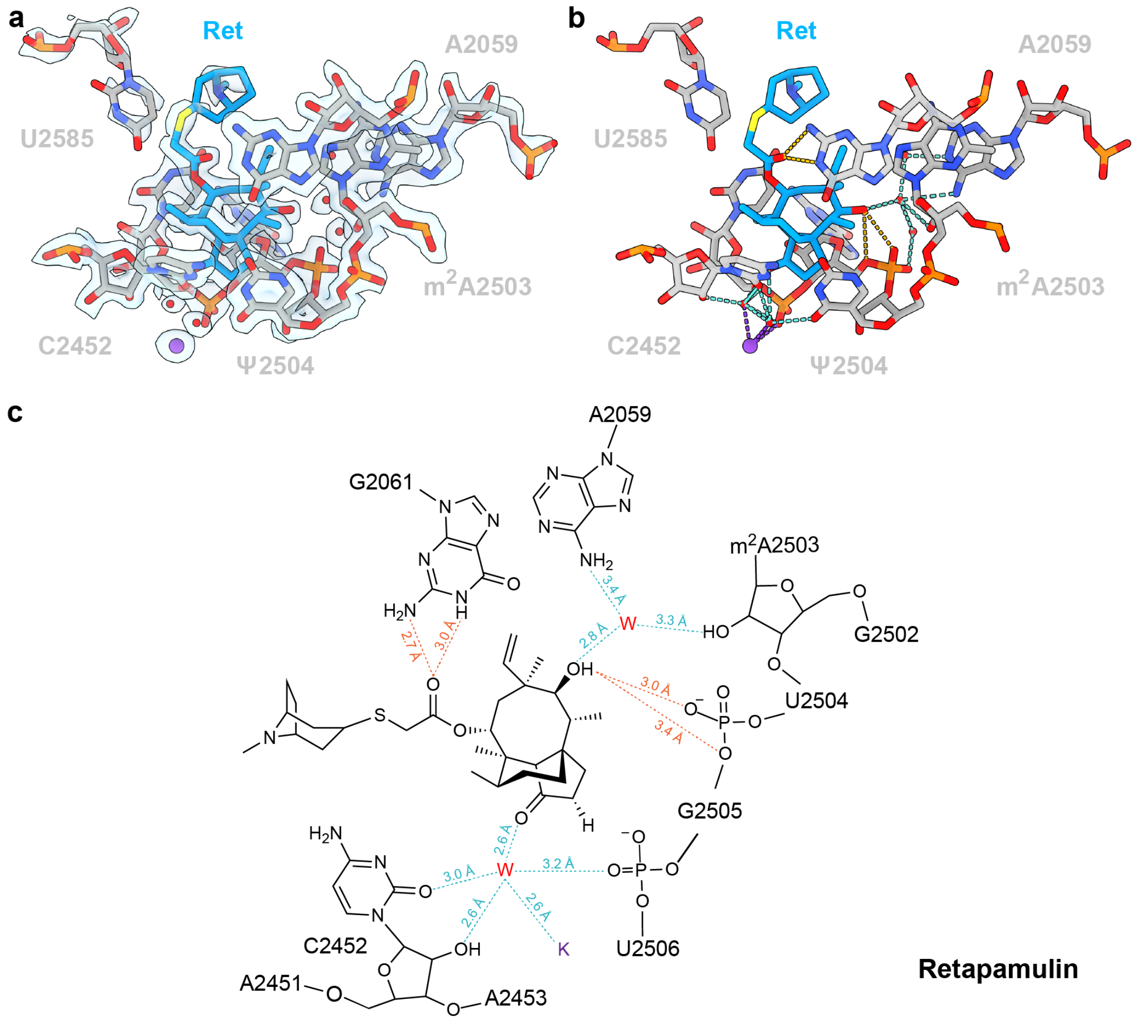

Supplementary Fig. 15 | Interaction of retapamulin on the LSU. a, cryo-EM density (transparent surface) for drug and nucleotides (not for solvent) with molecule model for retapamulin (blue), 23S rRNA nucleotides (grey) and water molecules (red spheres). **b,** same view as (**a**) without density but with dashed lines indicating direct (orange) or indirect (blue) water-mediated interactions. **c,** schematic representation of the indirect water (blue) and direct (orange) interactions between retapamulin and the LSU, with water (red W) and putative K+ ion (purple K) indicated. The putative K+ ion was assigned based on [1].

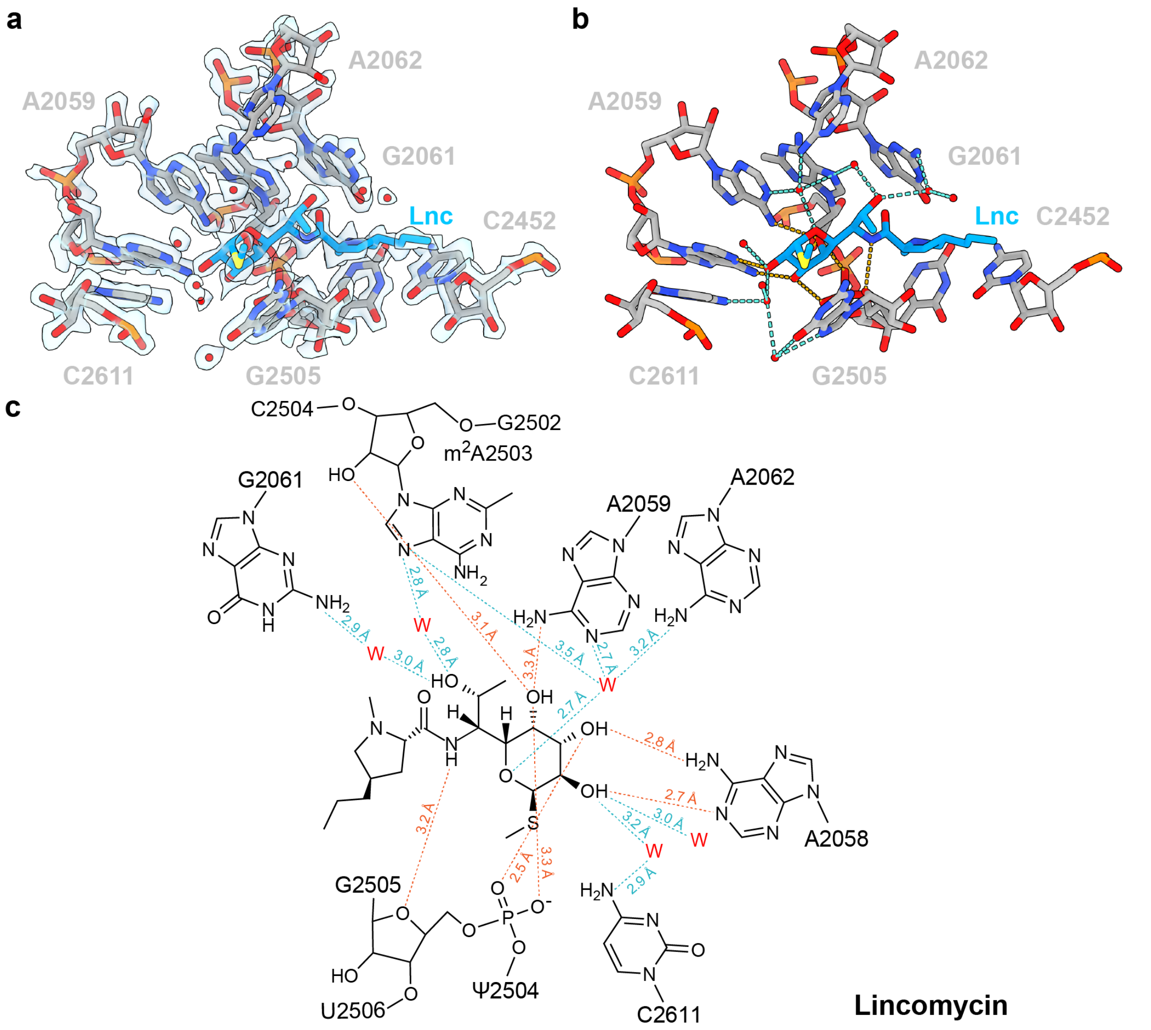

Supplementary Fig. 16 | Interaction of lincomycin on the LSU. a, cryo-EM density (transparent surface) for drug and nucleotides (not for solvent) with molecule model for lincomycin (blue), 23S rRNA nucleotides (grey) and water molecules (red spheres). **b,** same view as (**a**) without density but with dashed lines indicating direct (orange) or indirect (blue) water-mediated interactions. **c,** schematic representation of the indirect water (blue) and direct (orange) interactions between lincomycin and the LSU.

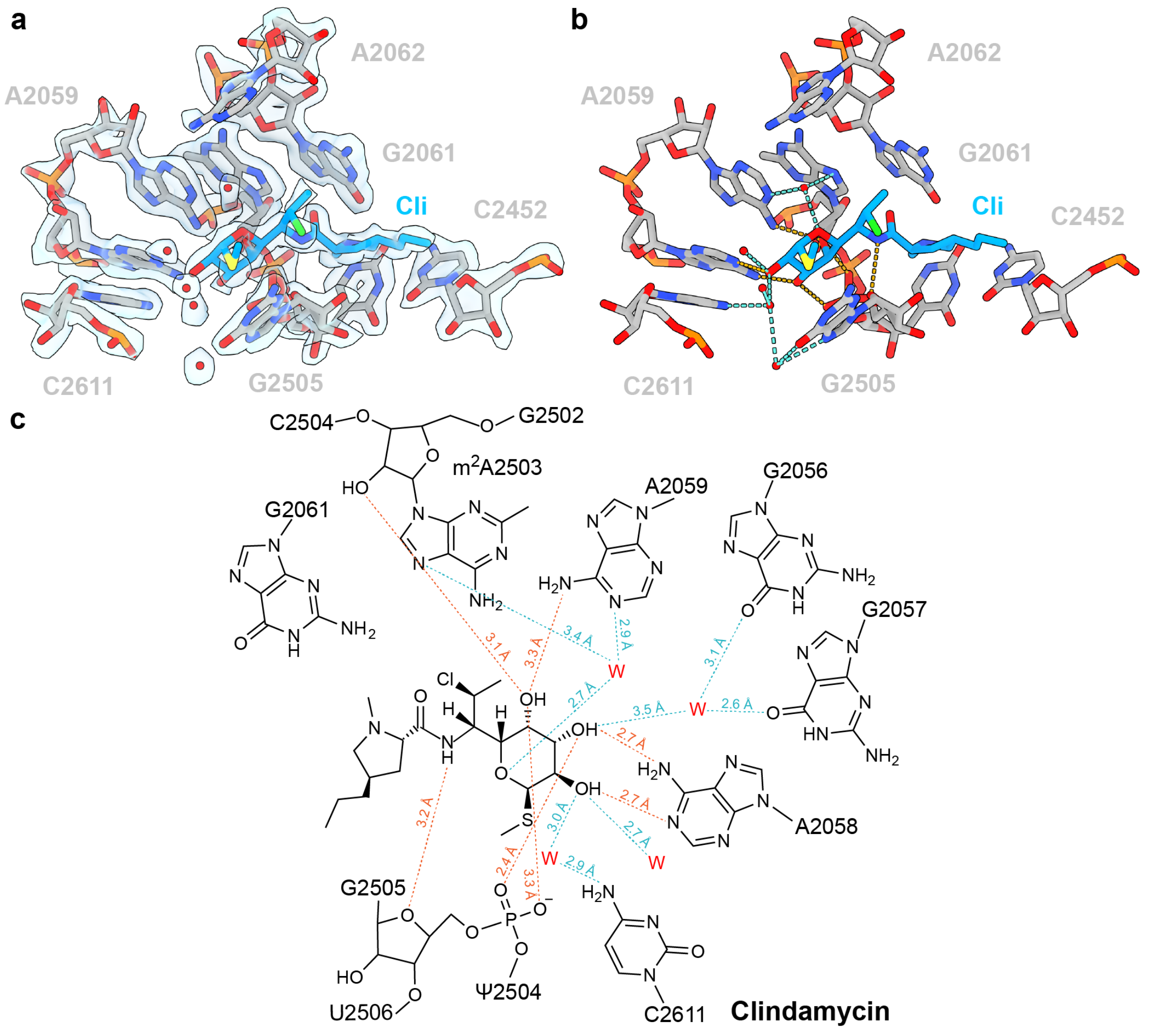

Supplementary Fig. 17 | Interaction of clindamycin on the LSU. a, cryo-EM density (transparent surface) for drug and nucleotides (not for solvent) with molecule model for clindamycin (blue), 23S rRNA nucleotides (grey) and water molecules (red spheres). **b,** same view as (**a**) without density but with dashed lines indicating direct (orange) or indirect (blue) water-mediated interactions. **c,** schematic representation of the indirect water (blue) and direct (orange) interactions between clindamycin and the LSU.

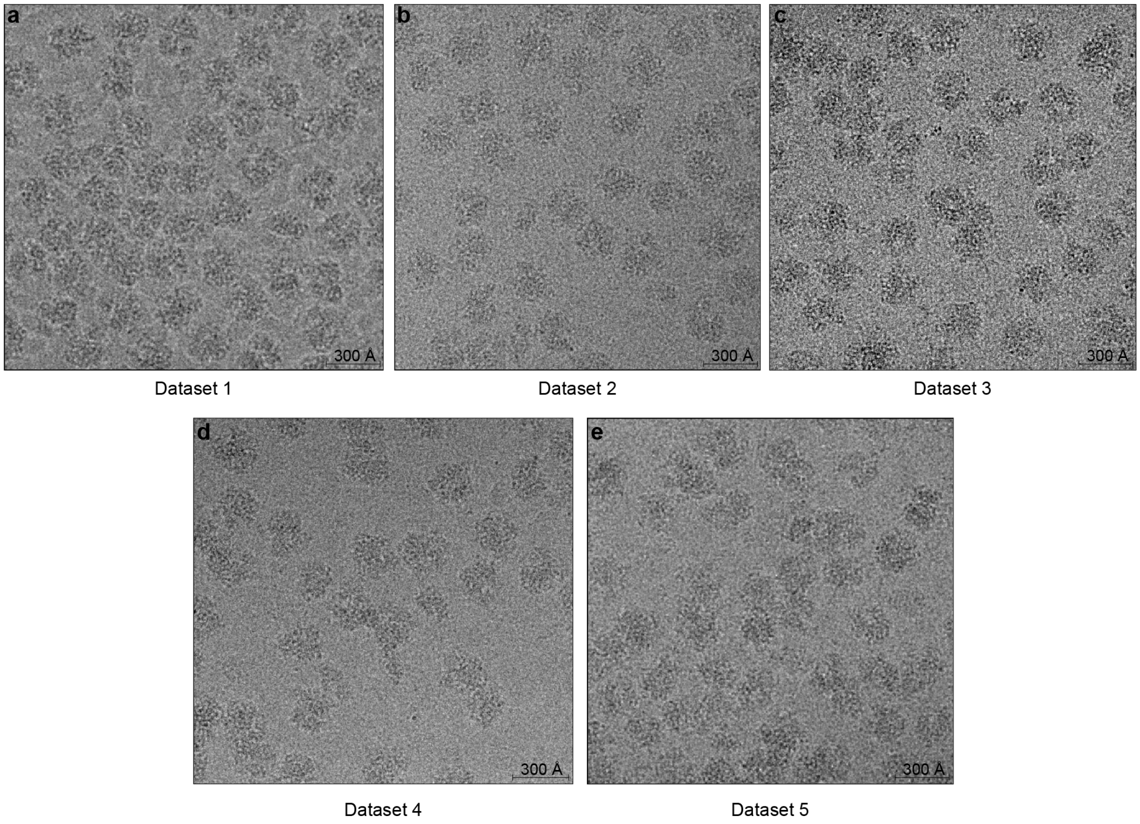

Supplementary Fig. 18 | Representative cryo-EM micrographs. a-e, Representative cryo-EM micrographs for (a) Datasets 1, (b) Dataset 2, (c) Dataset 3, (d) Dataset 4, and (e) Dataset 5. The scale is given in the bottom right hand corner for each micrograph.

Supplementary Table 1: Summary of antibiotic-ribosome structures

Antibiotic	Particle	Organism	Resolution	PDB ID	Reference
Lincomycin	50S	*S. aureus*	3.66 Å	5HKV	[2]
Clindamycin	50S	*H. marismortui*	3.0 Å	1YJN	[3]
Clindamycin	70S	*E. coli*	3.29 Å	4V7V	[4]
Clindamycin	50S	*D. radiodurans*	3.1 Å	1JZX	[5]
Iboxamycin	70S	*T. thermophilus*	2.5 Å	7RQ8	[6]
Tiamulin	50S	*D. radiodurans*	3.5 Å	1XBP	[7]
Tiamulin	50S	*H. marismortui*	3.2 Å	3G4S	[8]
Lefamulin	50S	*S. aureus*	3.55 Å	5HL7	[9]
Retapamulin	50S	*D. radiodurans*	3.66 Å	2OGO	[10]
Viomycin	70S	*E. coli*	3.29 Å	6LKQ	[11]
Viomycin	70S	*T. thermophilus*	3.0 Å	4V7L	[12]
Viomycin	70S	*E. coli*	2.9 Å	4V9O	[13]
Viomycin	70S	*E. coli*	3.2 Å	7ST7	[14]
Capreomycin	70S	*T. thermophilus*	3.45 Å	4V7M	[12]
Capreomycin	70S	*M. tuberculosis*	4.0 Å	5V93	[15]
Avilamycin	50S	*D. radiodurans*	3.43 Å	5JVG	[16]
Avilamycin	70S	*E. coli*	3.9 Å	5KCR	[17]
Evernimicin	50S	*D. radiodurans*	3.58 Å	5JVH	[16]
Evernimicin	70S	*E. coli*	3.9 Å	5KCS	[17]
Tetracycline	30S	*T. thermophilus*	4.5 Å	1I97	[18]
Tetracycline	30S	*T. thermophilus*	3.4 Å	1HNW	[19]
Tetracycline	70S	*T. thermophilus*	3.3 Å	4V9A	[20]
Tetracycline	70S	*E. coli*	2.8 Å	5J5B	[21]
Tigecycline	30S	*T. thermophilus*	3.4 Å	4YHH	[22]
Tigecycline	70S	*T. thermophilus*	3.1 Å	4V9B	[20]
Tigecycline	70S	*T. thermophilus*	2.96 Å	5J91	[21]
Eravacycline	70S	*A. baumannii*	2.55 Å	7M4W	[23]
Streptomycin	30S	*T. thermophilus*	3.0 Å	1FJG	[24]
Streptomycin	30S	*T. thermophilus*	3.3 Å	4DR6	[25]
Streptomycin	30S	*T. thermophilus*	3.35 Å	4JI1	[26]
Streptomycin	28S	Human mitorib.	2.4 Å	7P2E	[27]
Spectinomycin	30S	*T. thermophilus*	3.0 Å	1FJG	[24]
Spectinomycin	70S	*E. faecalis*	2.9 Å	7P7Q	[28]
Spectinomycin	70S	*E. coli*	3.5 Å	4V57	[29]
Spectinomycin	70S	*E. coli*	2.54 Å	7N2V	[30]
Apramycin	70S	*E. coli*	3.5 Å	4AQY	[31]
Apramycin	70S	*E. coli*	2.35 Å	7PJS	[32]
Apramycin	70S	*E. coli*	3.1 Å	7PJV	[32]
Gentamicin	70S	*E. coli*	3.54 Å	4V9C	[29]
Gentamicin	30S	*T. thermophilus*	3.28 Å	4LF9	-
Hygromycin B	30S	*T. thermophilus*	3.3 Å	1HNZ	[19]
Hygromycin B	70S	*E. coli*	3.5 Å	4V64	[33]

Hygromycin B	30S	*T. thermophilus*	3.7 Å	4LFA	[34]
Kasugamycin	30S	*T. thermophilus*	3.4 Å	2HHH	[35]
Kasugamycin	70S	*E. coli*	3.5 Å	4V4H	[19]
Paromomycin	30S	*T. thermophilus*	3.0 Å	1FJG	[24]
Paromomycin	70S	*T. thermophilus*	2.8 Å	4V51	[36]
Paromomycin	70S	*L. donovani*	2.5 Å	6AZ3	[37]
Paromomycin	70S	*E. coli*	1.98 Å	7K00	[38]

References

1 Rozov, A. *et al.* Importance of potassium ions for ribosome structure and function revealed by long-wavelength X-ray diffraction. *Nat Commun* **10**, 2519 (2019).

2 Matzov, D. *et al.* Structural insights of lincosamides targeting the ribosome of Staphylococcus aureus. *Nucleic Acids Res* **45**, 10284-10292 (2017).

3 Tu, D., Blaha, G., Moore, P. & Steitz, T. Structures of MLSBK antibiotics bound to mutated large ribosomal subunits provide a structural explanation for resistance. *Cell* **121**, 257-270 (2005).

4 Dunkle, J. A., Xiong, L., Mankin, A. S. & Cate, J. H. Structures of the Escherichia coli ribosome with antibiotics bound near the peptidyl transferase center explain spectra of drug action. *Proc Natl Acad Sci U S A* **107**, 17152-17157 (2010).

5 Schlünzen, F. *et al.* Structural basis for the interaction of antibiotics with the peptidyl transferase centre in eubacteria. *Nature* **413**, 814-821 (2001).

6 Mitcheltree, M. J. *et al.* A synthetic antibiotic class overcoming bacterial multidrug resistance. *Nature* **599**, 507-512 (2021).

7 Schlunzen, F., Pyetan, E., Fucini, P., Yonath, A. & Harms, J. Inhibition of peptide bond formation by pleuromutilins: the structure of the 50S ribosomal subunit from Deinococcus radiodurans in complex with tiamulin. *Mol. Microbiol.* **54**, 1287-1294 (2004).

8 Gurel, G., Blaha, G., Moore, P. B. & Steitz, T. A. U2504 determines the species specificity of the A-site cleft antibiotics: the structures of tiamulin, homoharringtonine, and bruceantin bound to the ribosome. *J Mol Biol* **389**, 146-156 (2009).

9 Eyal, Z. *et al.* A novel pleuromutilin antibacterial compound, its binding mode and selectivity mechanism. *Scientific reports* **6**, 39004 (2016).

10 Davidovich, C. *et al.* Induced-fit tightens pleuromutilins binding to ribosomes and remote interactions enable their selectivity. *Proc Natl Acad Sci USA* **104**, 4291-4296 (2007).

11 Zhang, L. *et al.* The structural basis for inhibition of ribosomal translocation by viomycin. *Proc Natl Acad Sci U S A* **117**, 10271-10277 (2020).

12 Stanley, R. E., Blaha, G., Grodzicki, R. L., Strickler, M. D. & Steitz, T. A. The structures of the anti-tuberculosis antibiotics viomycin and capreomycin bound to the 70S ribosome. *Nat. Struct. Mol. Biol.* **17**, 289-293 (2010).

13 Pulk, A. & Cate, J. H. Control of ribosomal subunit rotation by elongation factor G. *Science* **340**, 1235970 (2013).

14 Carbone, C. E. *et al.* Time-resolved cryo-EM visualizes ribosomal translocation with EF-G and GTP. *Nat Commun* **12**, 7236 (2021).
15 Yang, K. *et al.* Structural insights into species-specific features of the ribosome from the human pathogen Mycobacterium tuberculosis. *Nucleic Acids Res* **45**, 10884-10894 (2017).
16 Krupkin, M. *et al.* Avilamycin and evernimicin induce structural changes in rProteins uL16 and CTC that enhance the inhibition of A-site tRNA binding. *Proc Natl Acad Sci U S A* **113**, E6796-E6805 (2016).
17 Arenz, S. *et al.* Structures of the orthosomycin antibiotics avilamycin and evernimicin in complex with the bacterial 70S ribosome. *Proc. Natl. Acad. Sci. USA* **113**, 7527-7532 (2016).
18 Pioletti, M. *et al.* Crystal structures of complexes of the small ribosomal subunit with tetracycline, edeine and IF3. *EMBO J.* **20**, 1829-1839 (2001).
19 Brodersen, D. E. *et al.* The structural basis for the action of the antibiotics tetracycline, pactamycin, and hygromycin B on the 30S ribosomal subunit. *Cell* **103**, 1143-1154 (2000).
20 Jenner, L. *et al.* Structural basis for potent inhibitory activity of the antibiotic tigecycline during protein synthesis. *Proc Natl Acad Sci U S A* **110**, 3812-3816 (2013).
21 Cocozaki, A. I. *et al.* Resistance mutations generate divergent antibiotic susceptibility profiles against translation inhibitors. *Proc Natl Acad Sci U S A* **113**, 8188-8193 (2016).
22 Schedlbauer, A. *et al.* Structural characterization of an alternative mode of tigecycline binding to the bacterial ribosome. *Antimicrob Agents Chemother* **59**, 2849-2854 (2015).
23 Zhang, Z., Morgan, C. E., Bonomo, R. A. & Yu, E. W. Cryo-EM Determination of Eravacycline-Bound Structures of the Ribosome and the Multidrug Efflux Pump AdeJ of Acinetobacter baumannii. *mBio* **12**, e0103121 (2021).
24 Carter, A. P. *et al.* Functional insights from the structure of the 30S ribosomal subunit and its interactions with antibiotics. *Nature* **407**, 340-348 (2000).
25 Demirci, H. *et al.* A structural basis for streptomycin-induced misreading of the genetic code. *Nat Commun* **4**, 1355 (2013).
26 Demirci, H. *et al.* The central role of protein S12 in organizing the structure of the decoding site of the ribosome. *RNA* **19**, 1791-1801 (2013).
27 Khawaja, A. *et al.* Distinct pre-initiation steps in human mitochondrial translation. *Nat Commun* **11**, 2932 (2020).

28 Crowe-McAuliffe, C. *et al.* Structural basis for PoxtA-mediated resistance to phenicol and oxazolidinone antibiotics. *Nat Commun* **13**, 1860 (2022).
29 Borovinskaya, M. A. *et al.* Structural basis for aminoglycoside inhibition of bacterial ribosome recycling. *Nat Struct Mol Biol* **14**, 727-732 (2007).
30 Rundlet, E. J. *et al.* Structural basis of early translocation events on the ribosome. *Nature* **595**, 741-745 (2021).
31 Matt, T. *et al.* Dissociation of antibacterial activity and aminoglycoside ototoxicity in the 4-monosubstituted 2-deoxystreptamine apramycin. *Proc Natl Acad Sci U S A* **109**, 10984-10989 (2012).
32 Petrychenko, V. *et al.* Structural mechanism of GTPase-powered ribosome-tRNA movement. *Nat Commun* **12**, 5933 (2021).
33 Borovinskaya, M. A., Shoji, S., Fredrick, K. & Cate, J. H. Structural basis for hygromycin B inhibition of protein biosynthesis. *RNA* **14**, 1590-1599 (2008).
34 Schluenzen, F. *et al.* The antibiotic kasugamycin mimics mRNA nucleotides to destabilize tRNA binding and inhibit canonical translation initiation. *Nat. Struct. Mol. Biol.* **13**, 871-878 (2006).
35 Schuwirth, B. S. *et al.* Structural analysis of kasugamycin inhibition of translation. *Nat Struct Mol Biol* **13**, 879-886 (2006).
36 Selmer, M. *et al.* Structure of the 70S ribosome complexed with mRNA and tRNA. *Science* **313**, 1935-1942 (2006).
37 Shalev-Benami, M. *et al.* Atomic resolution snapshot of Leishmania ribosome inhibition by the aminoglycoside paromomycin. *Nat Commun* **8**, 1589 (2017).
38 Watson, Z. L. *et al.* Structure of the bacterial ribosome at 2 A resolution. *eLife* **9** (2020).
